# Patient satisfaction after conversion from warfarin to direct oral anticoagulants for patients on extended duration of anticoagulation for venous thromboembolism – The SWAN Study

**DOI:** 10.1371/journal.pone.0234048

**Published:** 2020-06-04

**Authors:** Thomas Hendriks, Scott McGregor, Shilpa Rakesh, Julie Robinson, Kwok M. Ho, Ross Baker

**Affiliations:** 1 Perth Blood Institute, Hollywood Private Hospital, Perth, Western Australia, Australia; 2 Western Australian Centre for Thrombosis and Haemostasis, Murdoch University, Murdoch, Western Australia, Australia; 3 Department of Intensive Care Medicine, Royal Perth Hospital, Perth, Western Australia, Australia; 4 School of Veterinary & Life Sciences, Murdoch University, Murdoch, Western Australia, Australia; Inselspital Universitatsspital Bern, SWITZERLAND

## Abstract

**Background:**

Warfarin is an anticoagulant medication proven effective in the initial treatment and secondary prevention of venous thromboembolism. Anti-Xa direct oral anticoagulants are alternatives to warfarin; however there is limited data assessing satisfaction after switching from warfarin to an anti-Xa direct oral anticoagulant in patients for treatment of venous thromboembolism.

**Objectives:**

To assess medication satisfaction in patients requiring anticoagulation for venous thromboembolism after conversion from warfarin to an anti-Xa direct oral anticoagulant.

**Methods:**

A retrospective cohort study with prospective assessment of satisfaction and review of adverse events following anti-Xa direct oral anticoagulant replacement of warfarin for treatment of venous thromboembolism. Out of 165 patients who had switched from warfarin to rivaroxaban or apixaban from an outpatient haematology practice, 126 patients consented for a survey of patient’s relative satisfaction of anti-Xa direct oral anticoagulant therapy compared with previous warfarin therapy using the Anti-Clot Burden and Benefits Treatment Scale and SWAN Score.

**Results:**

The mean Anti-Clot Burden and Benefits and SWAN Score was 93% (56/60) and 83% (24.8/30) respectively reflecting high satisfaction with anti-Xa direct oral anticoagulants. 120 patients stated preference for anti-Xa direct oral anticoagulants over warfarin. Leading perceptions driving this was the reduction in frequency of medical contact and fewer bleeding side effects. Thirteen patients (10.3%) experienced an adverse event after the anti-Xa direct oral anticoagulant switch (majority were non-major bleeding) but most remained on anti-Xa direct oral anticoagulant treatment after management options were implemented with continued high satisfaction scores.

**Conclusions:**

Patient satisfaction with anti-Xa direct oral anticoagulant therapy for the treatment and prevention of venous thromboembolism after switching from warfarin in routine clinical practice appeared high. Improved patient convenience including reduced frequency of medical contact and fewer unpredictable side effects were perceived as significant advantages of anti-Xa direct oral anticoagulants compared to warfarin.

## Introduction

Atrial fibrillation (AF), venous thromboembolism (VTE) including deep vein thrombosis (DVT) and pulmonary embolism (PE) and prosthetic heart valves remain the most common indications for warfarin therapy. Warfarin has been in use for over 60 years and was the 41^st^ highest volume drug in Australia over a 12-month period from 2016 to 2017. [1] However, as of 2012, two anti-Xa direct oral anticoagulants–DOACs (apixaban and rivaroxaban) became available for the treatment and prevention of VTE. [2] The DOACs are growing in popularity among physicians prescribing anticoagulants, and both apixaban and rivaroxaban ranked in the top four medications undergoing the most significant volume change over a one-year period from 2016 to 2017 in Australia. [1] There are several benefits of these DOACs over the established therapy warfarin. A major benefit of the DOACs is their predictable dose-related response, which removes the need for INR monitoring with warfarin use. Other benefits include fewer food and drug interactions, a wider therapeutic window and a rapid onset of action. [3] These DOACs also have comparable, if not better, safety and efficacy profiles compared to warfarin in the treatment of thromboembolic diseases.

The large pivotal studies in DVT and PE of over 12000 patients demonstrated the non-inferior efficacy of rivaroxaban and apixaban in the acute treatment of sub-massive PE and/or DVT when compared to standard therapy with initial low molecular weight heparin and followed by warfarin. [4–6] Rivaroxaban and apixaban were associated with equal or less major bleeding events than warfarin for the treatment of acute VTE and less intracranial haemorrhage in AF studies. [4–7] Patients with high risk of recurrence of VTE such as those suffering from an unprovoked VTE, have the choice of long-term risk reduction with dose reduced DOAC anticoagulation for secondary prevention of recurrent VTE with a low risk of major bleeding. [8, 9]

Patient satisfaction with medication is an important determinant of long-term patient compliance. Although the potential benefits of DOACs over warfarin are apparent from the clinical trials, there is limited data assessing patient satisfaction with DOACs compared to warfarin in real world clinical practice. [10] We examined patients who were switched from long-term warfarin to DOACs, and measured determinants of patient satisfaction, and specifically their experience of DOACs versus warfarin.

## Materials and methods

**Fig 1:**
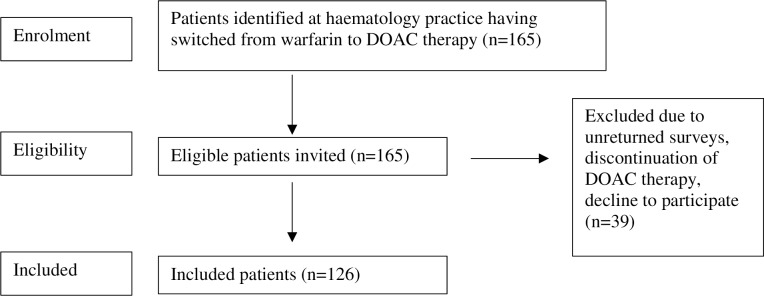
Study patient flow chart.

This cohort study was conducted at an outpatient specialist haematology practice (Perth Blood Institute) at Hollywood Private Hospital in Perth, Western Australia. Apixaban and rivaroxaban are the only approved DOACs in Australia for treatment of VTE. The study was approved by Hollywood Private Hospital Ethics Committee (HPH62) and registered with the ANZCTR (ACTRN12618000786291). During the study period, apixaban 5mg twice daily or rivaroxaban 20mg daily was approved for VTE treatment and only apixaban 2.5mg twice daily for extended VTE prophylaxis. The laboratory and patients’ physician supervised warfarin dosing according to INR. Out of 165 VTE identified patients on prolonged anticoagulation with warfarin who were identified to be suitable to change to a DOAC, written informed consent was obtained from 126 patients. Inclusion criteria for invitation included adult (>18yrs), patients that were switched from warfarin to rivaroxaban or apixaban (and currently on one of these two medications), between August 2011 and January 2017 for the treatment and prevention of recurrence of VTE (Fig 1). Patients were excluded if they discontinued DOAC therapy during the study period (August 2011 –January 2017) or if they switched back to warfarin anticoagulation therapy.

Information sheets, survey/questionnaire and withdrawal of consent forms were sent to the patients. If the patients did not return the survey within the four-week allotted time, a courtesy call to the patients was made by an investigator (TH) to invite their participation. Data collection occurred between February 2016 and January 2017, through the use of electronic patient files (medical records) and through the questionnaires delivered either by mail, over the phone or in person with patients whom consented to participate. Data was recorded at the Perth Blood Institute on a secure network database on password and firewall protected computers. Only approved haematology practice staff and associates were able to access the database through a secure login. The data was de-identified once entered into the PBI database and all subsequent analysis was performed on the de-identified data.

Patient-reported satisfaction with DOAC therapy was prospectively assessed using the Anti-Clot Treatment Burden and Benefits Scale (ACTS) in addition to the newly developed questionnaire (SWAN score: Satisfaction with Warfarin and direct oral AnticoagulaNts) that directly compared patient’s satisfaction with warfarin to DOAC therapy. [11]

The ACTS is a well-established, self-administered, questionnaire that assesses satisfaction with anticoagulant treatment that can be used in patients with AF and VTE. Its reliability and validity for ACTS was examined in a large DVT treatment trial and was delivered among various cultures groups and validated against a similar patient-reported instrument of satisfaction (the Treatment Satisfaction Questionnaire for Medication version 2 –TSQM II). [11] Commonly used psychometric properties such as data acceptability, scaling, internal consistency and responsiveness were applied to the ACTS questionnaire and these results supported its use as a treatment satisfaction tool. [11] The questionnaire is made up of two main scores, Benefits score (linear scale) and Burdens Scores (inverse scale). The Benefits score consists of 3 questions assessing several domains including confidence in anticoagulation, reassurance of efficacy of anticoagulation and overall satisfaction with anticoagulation. Each question is scored out of five giving a total score out of 15. The Burdens score consists of 12 questions assessing several challenges associated with anticoagulation. These scores are also out of five and give a total score out of 60. Both the Benefits and Burdens scores out of five are scored by patients as follows: 1 = not at all, 2 = a little, 3 = moderately, 4 = quite a bit, 5 = extremely. The Burdens score is calculated on an inverse scale meaning that higher scores are indicative of greater satisfaction with anticoagulation. The Benefits score is calculated on a linear scale meaning higher scores are also indicative of greater satisfaction. As such the maximum for each of the Benefits and Burdens score which correlates with high satisfaction with treatment are 15/15 and 60/60 respectively. In addition, there are two overall impact scores: one positive and one negative that assess the impact anticoagulation has had on the patients’ lives, these are both scored out of five.

The specifically designed SWAN Survey score was developed for several reasons. In the absence of being able to deliver the ACTS to patients on warfarin prior to changing to DOACs, the SWAN survey allowed direct comparison of patient satisfaction after switching. Questions were designed to present some of the real-world practicalities, issues and/or burdens associated with anticoagulation. Despite not being a validated score, it offered some insight into the patient’s real-world subjective experiences and preferences for DOAC therapy compared to warfarin. The SWAN questionnaire comprised of a seven-item questionnaire asking participants to score their satisfaction out of five for DOAC vs. warfarin therapy (1 = much less satisfied, 2 = less satisfied, 3 = no change, 4 = more satisfied, 5 = much more satisfied). Questions sought comparisons between the anticoagulants with regards to side effects, reduced medical contact, dietary restriction, medication interactions, changes in travel, changes in cost, and overall satisfaction. Finally, the participants were asked to state which anticoagulation therapy they preferred and for what reasons (convenience, tolerance and effectiveness were all listed as pre-existing options).

### Statistical analysis

The differences in the ACTS and SWAN scores between those taking apixaban and rivaroxaban were analysed by Mann-Whitney test. Multivariate logistic regression was used to identify demographic and clinical factors that were significantly associated with the highest level of satisfaction–defined by an ACTS and SWAN score of 5—with the DOAC therapy and the comparison between DOAC and warfarin, respectively. Sample size was estimated by allowing 10 subjects (with a SWAN score of 5) per predictor, assuming at least 30% of the subjects would give a SWAN score 5, and a total of 5 predictors were analysed in the multivariate analyses. All analyses were conducted by SPSS for Windows (version 24.0, 2017, IBM, USA), a p value <0.05 was taken as significant, and Bonferroni adjustment was not used for multiple statistical testing.

## Results

165 patients were identified as being treated with anti-Xa DOAC for the extended treatment of VTE (rivaroxaban n = 119, apixaban n = 46) after switching from warfarin between August 2011 and January 2017. Patients taking rivaroxaban received 20mg once daily and the dose of apixaban was either 2.5mg twice daily or 5mg twice daily dependent on clinician and patient choice, indication (subsequent or extended treatment of VTE), weight, renal function and age. Thirty-one patients did not return surveys, five discontinued DOAC therapy for unknown reasons, and three patients declined to participate in the study. The remaining 126 patients formed the basis of the study. There were complete survey response rates for both the ACTS and SWAN survey scores, however four patients did not indicate which anticoagulation therapy they preferred and 15 indicated a preference without listing a reason.

The median age was 64 years (range: 21–84 years) and overall 66% were female (75% apixaban, 47% rivaroxaban). The median duration of warfarin therapy for the whole cohort prior to switching to an anti-Xa DOAC was 5.4 years (range: less than 1 to 33 years). The most common indications for switching from warfarin to a DOAC included intolerance/side effects with warfarin, INR instability and reduced monitoring requirements.

ACTS scores among the whole cohort were high (Table 1). The median Burden and Benefit ACTS scores across the whole cohort were 56 (range: 38–60) and 12.7 (range: 3–15), respectively, reflective of excellent satisfaction with the DOAC (inverse scale scoring system for Burdens score). The median Positive Impact score in the cohort was 4.2 (range: 1–5), confirming DOAC therapy’s overall good acceptance by the participants. There was no statistically significant difference between the mean ACTS satisfaction scores in patients treated with rivaroxaban or apixaban (p = 0.15). Female (p = 0.014) and a long duration of warfarin treatment prior to switching to DOAC (p = 0.049) were the only two predictors of having good acceptance of the DOAC (ACTS score = 5) (Table 2).

**Table 1:** Differences Anti-Clot Burden and Benefits Treatment Scale (ACTS) and SWAN score (on satisfaction compared to warfarin) between those who were treated with apixaban and rivaroxaban (mean and range). Higher scores reflect greater satisfaction/less burden.

Variable	Apixaban (n = 40)	Rivaroxaban (n = 86)	P value^#^
***ACTS score*: *-***			
Perceived burden score (12 questions, range 12–60)	57 (52–59)	57 (55–59)	0.334
Overall negative impact (Range 1–5)	5 (4–5)	5 (4–5)	0.415
Perceived benefit score (3 questions, range 3–15)	13 (10–15)	13 (12–15)	0.349
Overall positive impact (Range 1–5)	4 (4–5)	4 (4–5)	0.505
***SWAN score*: *-***			
Total score (6 questions, range 6–30)	24 (21–28)	25 (23–28)	0.125
Overall satisfaction (1 question, range 1–5)	4.4 (4–5)	4.8 (5–5)	*0*.*004*

^#^ Mann-Whitney test.

**Table 2:** Multivariate analyses showing the relationships between demographic or other factors and (a) an extremely good overall positive impact Anti-Clot Burden and Benefits Treatment Scale (ACTS = 5), (b) a feeling of much more satisfied with direct oral anticoagulant than warfarin (SWAN overall satisfactory score = 5).

Variables	Odds ratio (95% confidence interval)	P value
ACTS overall positive impact score = 5:*		
Age *(per year increment)*	0.97 (0.94–1.01)	0.095
Female	2.80 (1.24–6.36)	*0*.*014*
Apixaban	0.70 (0.29–1.69)	0.70 (0.29–1.69)
*(compared to rivaroxaban)*		
Duration of warfarin	1.06 (1.01–1.13)	*0*.*049*
*(per year increment)*		
No bleeding complications	No bleeding complications	0.775
SWAN overall satisfactory score = 5:**		
Age *(per year increment)*	0.99 (0.96–1.03)	0.612
Female	2.05 (0.84–5.01)	0.117
Apixaban	0.29 (0.11–0.73)	*0*.*009*
*(compared to rivaroxaban)*		
Duration of warfarin		0.180
*(per year increment)*		
No bleeding complications	2.48 (0.41–15.13)	0.326

*The Hosmer-Lemeshow Chi Square and Nagelkerke R^2^ of this model were 10.1 (p = 0.257) and 0.136, respectively.

**The Hosmer-Lemeshow Chi Square and Nagelkerke R^2^ of this model were 5.2 (p = 0.733) and 0.135, respectively.

**Fig 2:**
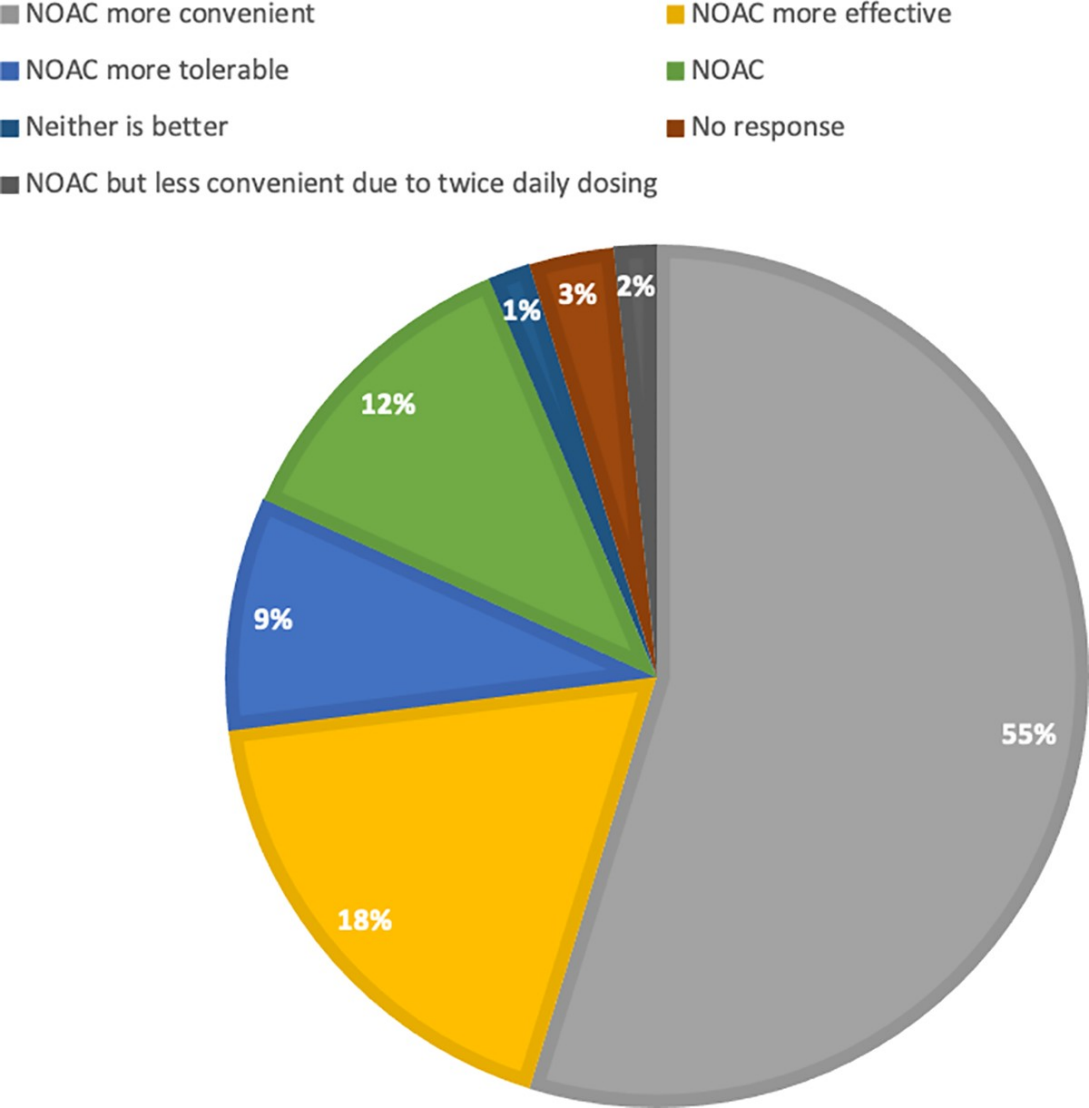
Patient preference for anticoagulation and rationale.

Overall, patients’ satisfaction with DOAC (as compared to warfarin) was high; with mean SWAN scores across all seven domains for both apixaban and rivaroxaban were above three. A vast majority of patients (95%) stated preference for the DOAC over warfarin because of perceptions of convenience, effectiveness and tolerability (Fig 2). Two patients expressed concern regarding the lack of availability of an antidote for their rivaroxaban; however, they were still more satisfied with DOAC overall compared to warfarin. Rivaroxaban was associated with higher overall satisfaction compared to apixaban (p = 0.004), and this remained unchanged in multivariable analysis (odd ratio for SWAN score of 5: apixaban versus rivaroxaban 0.29, 95%CI 0.11–0.73; p = 0.009)(Table 2).

Thirteen patients (10.3%) had documented evidence of an adverse event (AE) related to DOAC therapy (rivaroxaban = 12, apixaban = 1, Table 3). Seven patients experienced clinically significant non-major bleeding-related AEs, all occurring on rivaroxaban. Menorrhagia was the most common of these (n = 5) managed with placement of intra-uterine device (Mirena^®^) (n = 3) or reduced anticoagulation dose to apixaban 2.5mg twice daily (n = 2) without further AEs. Two patients had thrombotic complications; one patient suffered a transient ischaemic attack and was commenced on aspirin in addition to the rivaroxaban therapy (20mg once daily). The other patient suffered from a gastrocnemius vein thrombosis whilst on apixaban (5mg twice daily) and was temporarily switched back to warfarin then subsequently back to apixaban. Four patients suffered non-bleeding or thrombotic related AEs with rivaroxaban including itch, diarrhoea, and nausea and vomiting. These patients were subsequently switched to apixaban with no further events. Surprisingly of the 13 surveyed patients that experienced an AE, 12 (92.3%) maintained higher satisfaction with DOAC therapy compared to warfarin. The remaining patient who experienced recurrent haemarthrosis stated neither treatment was better.

**Table 3:** Adverse events after switching from warfarin to a DOAC with the SWAN mean overall satisfaction score and management.

Adverse Events Total N = 13	No.	Mean Overall Satisfaction Score with DOAC	Outcome
Bleeding	7		
• Menorrhagia	5	4.6 / 5	Managed with switching to dose reduced alternate DOAC (apixaban 2.5mg twice daily n = 2) or continued DOAC (rivaroxaban 20mg daily) and Mirena^®^ insertion (n = 3)
• Haemarthrosis	2	3.5 / 5	Managed with switching to dose reduced alternate DOAC (apixaban 2.5mg twice daily)
Thrombotic	2		
• Transient ischemic attack	1	5 / 5	Continued on DOAC in addition to aspirin
• Gastrocnemius vein thrombosis	1	4 / 5	Switched to warfarin temporarily then back to apixaban without further AEs
Other (itch, nausea, vomiting, diarrhoea)	4	5 / 5	All switched to apixaban without further AEs

## Discussion

The current study showed that most patients were satisfied with the switch from warfarin to DOAC therapy (either rivaroxaban or apixaban) for long-term VTE treatment. Female patients and those who had been treated with warfarin for an extended period of time were most likely to report a better satisfaction with the DOAC therapy. The perception of fewer side effects and requiring less medical contact or monitoring with the DOAC therapy were considered most important, and the majority of patients stated preference for the DOAC over warfarin because of convenience, effectiveness and tolerability.

DOAC is increasingly used as an anticoagulant to prevent stroke and thromboembolism because of the ageing population and the associated increased risk of AF and VTE with age. [12] In a recent study in Australia, around 72% of new anticoagulant prescriptions are for DOACs rather than warfarin among elderly veterans (median age 86). [12] The predictable dose related pharmacodynamics, fewer food and drug interactions, a wider therapeutic window and a rapid onset of action are making DOACs more popular, including patients with AF who lived in remote rural Australia. [13] Several of our patients specifically commented on the advantage of reduced medical contact and fewer restrictions to travel. Indeed, the ACTS scores for both DOACs were high and did not appear to differ. Nonetheless, some of our patients appeared to have a slightly better satisfaction after changing from warfarin to rivaroxaban than to apixaban. This result could be related to the fact that two patients reported that they disliked the twice daily apixaban dosing.

Unlike patients with AF where anticoagulation is prescribed for primary prevention of thromboembolic stroke, patients with VTE have already experienced an event and generally are highly motivated to prevent further recurrence. Both warfarin and DOACs have similar VTE risk reduction from the clinical studies, it would be expected that other factors such as safety, improved convenience and fewer side effects would drive the change in using one anticoagulant over the other. Recent large randomised controlled trials have demonstrated non-inferiority for DOAC compared to warfarin with overall similar or reduced major bleeding risk, including a reduction in intracranial bleeding. [4–7] Their efficacy in stroke prevention in AF and in the treatment/prevention of VTE has been extensively investigated, but only recently has there been initial investigation into patient satisfaction with DOAC therapy.

Patients’ satisfaction with both rivaroxaban and apixaban, as observed in this observational study (mean ACTS score 56/60) using real-world clinical practice data was consistent with other comparative studies on changing warfarin to rivaroxaban [14–16]. In addition, our study also examined patients requiring extended anticoagulation treatment of VTE for many years in contrast to most other studies that assessed satisfaction for shorter periods of time in newly diagnosed patients treated with DOACs for non-valvular AF or VTE. Patients’ satisfaction (and the ACTS score) with rivaroxaban compared to enoxaparin/warfarin therapy was reported by the EINSTEIN PE and DVT sub-studies. [14, 15] The mean Burden score with rivaroxaban was 55.4 compared to the enoxaparin/warfarin score of 51.9 (p<0.0001) in the DVT study. [14] Mean Burden scores were 55.2 and 52.6 (p<0.0001) in favour of rivaroxaban in the PE study. [15] Similarly favourable Benefit scores were evident in the rivaroxaban cohort compared to enoxaparin/warfarin in both studies, 11.9 vs. 11.4 (PE study) and 11.7 vs. 11.5 (DVT study). [14, 15] Similarly, in patients with non-valvular AF treated with a vitamin K antagonist (VKA) who switched to rivaroxaban, significant improvements in ACTS scores with Burden scores increased from 50.5 to 54.4 (p<0.001) and Benefit scores increased from 10.3 to 11.4 (p<0.0075) were reported in the recent XANTUS sub-study trial. [16] These results were supported by two further small-scale studies both favouring rivaroxaban to VKA therapy with improvement in both Burden and Benefit ACTS scores. [17, 18]. Our study confirmed overall high patient satisfaction as measured by the ACTS score with no difference found between either rivaroxaban or apixaban.

To consider further patients’ preferences, the SWAN score revealed specific real practical issues for patients changing to DOACs. The change in cost of DOAC appeared to contribute least towards overall satisfaction with a mean score of 3.6/5 across both apixaban and rivaroxaban cohorts. This score was still representative of overall heightened satisfaction with DOAC therapy compared to warfarin. The average increase in cost for DOAC therapy per prescription compared to warfarin is relatively small (depending on concession status) and regardless of this difference patient’s satisfaction was still enhanced as indicated by the SWAN scores. Furthermore, despite the discrepancy in patient cost because of the requirement for monthly repeat prescriptions, recent studies have suggested that DOAC therapy and in particular apixaban are in general cost-effective to the health system when compared to warfarin for stroke prevention in patients with non-valvular AF. [19–21]

After switching from warfarin to DOACs, clinically relevant non-major bleeding was the most common side effect that necessitated further management. This finding is consistent with the reports of menorrhagia in woman of childbearing age prescribed DOACs but with appropriate intervention, the DOAC can generally be continued for effective thrombosis risk reduction. [22] Several patients experienced non-haemostatic side effects (itch, nausea, vomiting diarrhoea) and a switch to the other DOAC overcame the drug specific side effect.

This study has several strengths and weaknesses. The strength of the study was that prospective real-world assessment of patient satisfaction was performed through the established ACTS score and a specifically designed SWAN Survey exploring how satisfied they were with either type of DOAC (apixaban or rivaroxaban) compared to previous warfarin therapy. Unlike other studies, the participants had already long-term experience with warfarin anticoagulation (average five years) prior to being switched to a DOAC and satisfaction was explored soon after change. The limitation is that the SWAN score has not been validated by other studies and therefore, its accuracy remains uncertain. Nonetheless, this study provides a real-world data on patients’ perception about their satisfaction with different types of anticoagulants. In this study, it was not possible to obtain the ACTS score prior to changing from warfarin to DOAC; our study produced similar ACTS scores to other anticoagulation satisfaction studies where mean burden scores ranged from 50 to 55 and benefit scores from 10.3 to 11.9. [9, 10, 12] No difference was found between rivaroxaban and apixaban treatment. The generalisability of our study is limited by its single centre design, small sample size and difference in baseline characteristics (including gender distribution) for those who were treated with rivaroxaban and apixaban. Finally, there was a potential selection bias in including only patients who had switched to DOAC therapy who might not be completely satisfied with the warfarin therapy in the first instance, thereby favouring their preferences for the DOACs.

## Conclusions

To our knowledge, the current SWAN Study is the first attempt in assessing satisfaction among patients who were treated with either rivaroxaban or apixaban for VTE in real world clinical practice. Patient satisfaction with DOAC therapy for the treatment and prevention of recurrence of VTE after switching from warfarin in routine clinical practice appears high, similar to those results reported about rivaroxaban in the DVT/PE and AF clinical trials. [9, 10, 12] The incidence of adverse events after switching to a DOAC appears low with menorrhagia being the most frequently observed bleeding-related adverse event. Improved patient convenience including reduced frequency of medical contact and fewer side effects, as demonstrated in this study, are perceived as the most significant advantages of DOAC treatment compared to warfarin. Further prospective studies comparing the long term ACTS scores between patients who are taking warfarin with self monitoring and DOACs and how the ACTS scores may change over time among those who switch between these two main types of anticoagulants are warranted.

## Supporting information

S1 Dataset Minimal data set file. (XLSX)
